# Nanostructured Hybrid Matrix Device: An Engineered Approach for Localized Photodynamic Therapy in 9L/lacZ Cell Models

**DOI:** 10.1021/acsomega.6c04974

**Published:** 2026-10-13

**Authors:** Jéssica A. R. Ambrósio, Janicy A. Carvalho, Letícia G. M. Pinto, Leandro J. Raniero, Vitor L. M. Marmo, Juliana G. Pinto, Tassia J. Martins, Juliana Ferreira-Strixino, Milton Beltrame Júnior, Andreza R. Simioni

**Affiliations:** 67655 Universidade do Vale do Paraiba, São José dos Campos 12244000, São Paulo, Brazil

## Abstract

Gliosarcoma treatment remains a challenge due to its aggressiveness and high recurrence rates. This study describes the development and characterization of a novel hybrid drug delivery system (DDS) for localized photodynamic therapy (PDT). The system consists of a PVA/gelatin hydrogel encapsulated with hydroxyapatite microspheres (meHAp) loaded with chloroaluminum phthalocyanine (ClAlPc). SEM and FTIR analysis confirmed the successful incorporation of meHAp into the porous 3D polymeric network. The addition of meHAp increased the matrix’s structural rigidity, adjusting the swelling degree and enabling a more controlled release of the photosensitizer (45% vs 94% in the control). Biological assays on 9L/lacZ gliosarcoma cells revealed high internalization and a 98% reduction in cell viability under PDT protocols. These results suggest that the meHAp-reinforced hydrogel system is a promising option for the localized treatment of brain tumors, providing a modified release of the photosensitizer.

## Graphical abstract

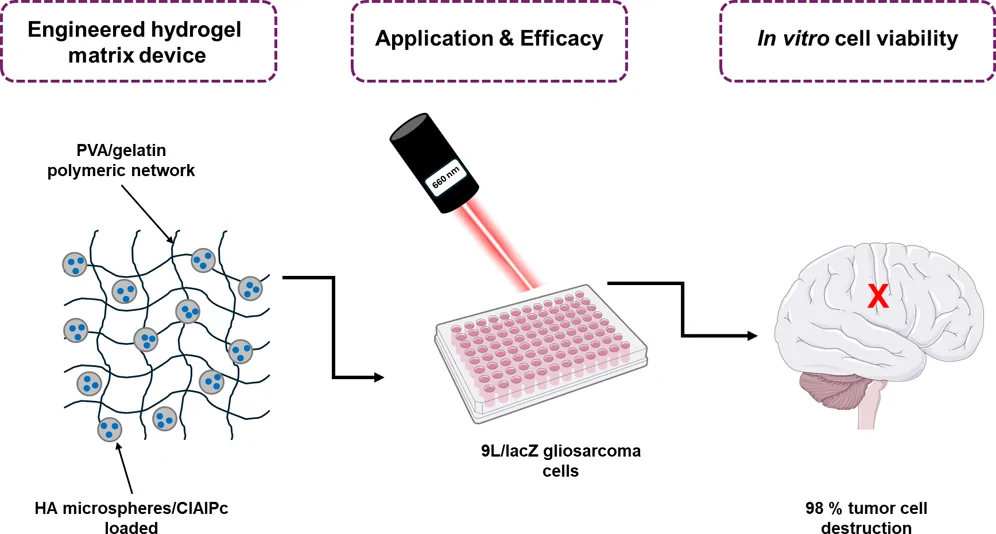

## 1 Introduction

Gliosarcoma is a rare and highly aggressive primary neoplasm of the central nervous system, characterized by a challenging clinical prognosis and high rates of local recurrence following surgical intervention. Despite advancements in neurosurgery and adjuvant therapies, the effective eradication of residual tumor cells in the surgical bed remains a significant hurdle, primarily due to the blood–brain barrier and the systemic toxicity of conventional drugs. In this scenario, localized therapeutic strategies based on implantable biomaterials have emerged as a promising Frontier. Among these, polymeric hydrogels stand out due to their high-water content and structural similarity to the extracellular matrix, offering a unique platform for the controlled delivery of therapeutic agents directly to the affected site.^1–3^

Photodynamic therapy (PDT) has gained prominence as a minimally invasive treatment modality that utilizes a photosensitizer (PS), oxygen, and light to induce localized tumor cell death. However, the clinical translation of potent photosensitizers, such as chloroaluminum phthalocyanine (ClAlPc), is often hindered by their hydrophobic nature and rapid clearance from the target site. To address these limitations, hydrogel-based delivery systems composed of biocompatible polymers like poly­(vinyl alcohol) (PVA) and Gelatin have been explored. While these matrices provide a suitable 3D environment, monolithic hydrogels often exhibit a pronounced ‘burst release’ effect, necessitating further structural modifications to achieve a truly sustained and controlled drug delivery profile.^4,5^

In this context, phthalocyanines are PSs with excellent ROS generation capacity and greater tissue penetration when irradiated with a irradiation source is 660 nm LEDs. Among them, chloroaluminum phthalocyanine (ClAlPc) is widely used in cancer therapy and exhibits a maximum absorption around 670 nm. However, like other variants in its class, ClAlPc is hydrophobic and undergoes aggregation processes in aqueous media.^6–8^

To enable the application of phthalocyanines as PSs in alternative treatments, a controlled-release system (DDS) is required. This model’s main objective is to address gaps in traditional treatment methods, including irregular release schedules, peak drug administration, high treatment costs, patient adherence, and medication-related side effects. DDS works by protecting the target drug and ensuring its delivery only to the target treatment area. This ensures higher delivery rates, reduced costs, and reduced application frequency, factors that can increase patient adherence and make treatment more effective.^9–11^

The type of DDS applied, as well as the materials used for its preparation, depend on the characteristics of the target treatment site and the characteristics of the materials to be loaded. Examples of systems include nanoparticles and microparticles, micelles, liposomes, and hydrogels. Due to their three-dimensional network structure, hydrogels can swell, allowing the chains to absorb and retain large amounts of water without dissolving. These materials also exhibit responsiveness to stimuli such as changes in pH and temperature, making them promising candidates for loading with PSs and other substances of interest. These systems can be formed from natural polymers, such as gelatin and alginate, or from synthetic polymers, such as poly­(vinyl alcohol) (PVA).^12–15^

Since the idea of DDS is to combine it with surgical procedures, it is important to use materials with properties that support patient adaptation and recovery. PVA is a synthetic polymer produced by the hydrolysis of polyvinyl acetate with biocompatibility and nontoxicity properties. Gelatin is a natural polymer derived from collagen and has high biocompatibility, nontoxicity, biodegradability, and low cost. It is widely used in various applications involving delivery systems. The mechanical strength of polymeric hydrogels can be increased by adding micro- or nanoparticle materials to the formed structure. Organic and inorganic materials such as graphene oxide, magnetic nanoparticles, and hydroxyapatite (HAp) have been used for this purpose. Hydroxyapatite is a ceramic material very similar to the human structure, with properties such as biocompatibility, osteoconduction, and nontoxicity.^16–18^

From a biomedical engineering perspective, the development of implantable scaffolds requires a precise balance between structural integrity and functional drug release kinetics. Traditional monolithic hydrogels often suffer from rapid degradation and a pronounced ‘burst release’ effect, which limits their long-term therapeutic efficacy in the postsurgical. In this context, the engineering of hybrid nanostructured systems—combining the flexibility of polymeric matrices like PVA and gelatin with the rigid, bioactive properties of ceramic components like hydroxyapatite—offers a sophisticated approach to modulate drug diffusion. These composite architectures act as a hierarchical barrier, where the inorganic microspheres serve as secondary reservoirs that protect the photosensitizer and ensure a sustained, localized delivery, directly addressing the clinical need for effective, site-specific photodynamic therapy in aggressive brain tumors.

To overcome the limitations of rapid drug diffusion and systemic toxicity, this work proposes the development of a hybrid nanostructured system. By integrating chloroaluminum phthalocyanine (ClAlPc)-loaded hydroxyapatite microspheres (meHAp) into a PVA/gelatin matrix, we engineered a dual-barrier delivery platform. This study aims to characterize the physicochemical properties of this hybrid hydrogel and, evaluate its photodynamic efficacy against the 9L/lacZ gliosarcoma cell line, offering a new perspective for the postoperative treatment of localized brain tumors. The authors highlight that the hydroxyapatite microspheres encapsulated with aluminum-chloro-phthalocyanine (meHApClAlPc) originate from a previous study by the research group (Ambrosio et al.^19^), which presents the general methodology and prior characterizations of the meHApClAlPc used in describing the dual-barrier system addressed in this article.

## 2 Results and Discussion

### 2.1 Morphological Characterization

SEM micrographs (Figure 1a,b) revealed a highly porous, interconnected 3D network within the PVA/gelatin hydrogels. The incorporation of meHAp microspheres resulted in a rougher surface morphology, in which inorganic particles were successfully embedded into the polymer walls. This heterogeneous architecture is crucial for biomedical applications, as the interfacial adhesion between the ceramic microspheres and the hydrogel matrix acts as physical reinforcement, potentially increasing the tortuosity of diffusion pathways for the encapsulated photosensitizer.^20–26^

**Figure 1:**
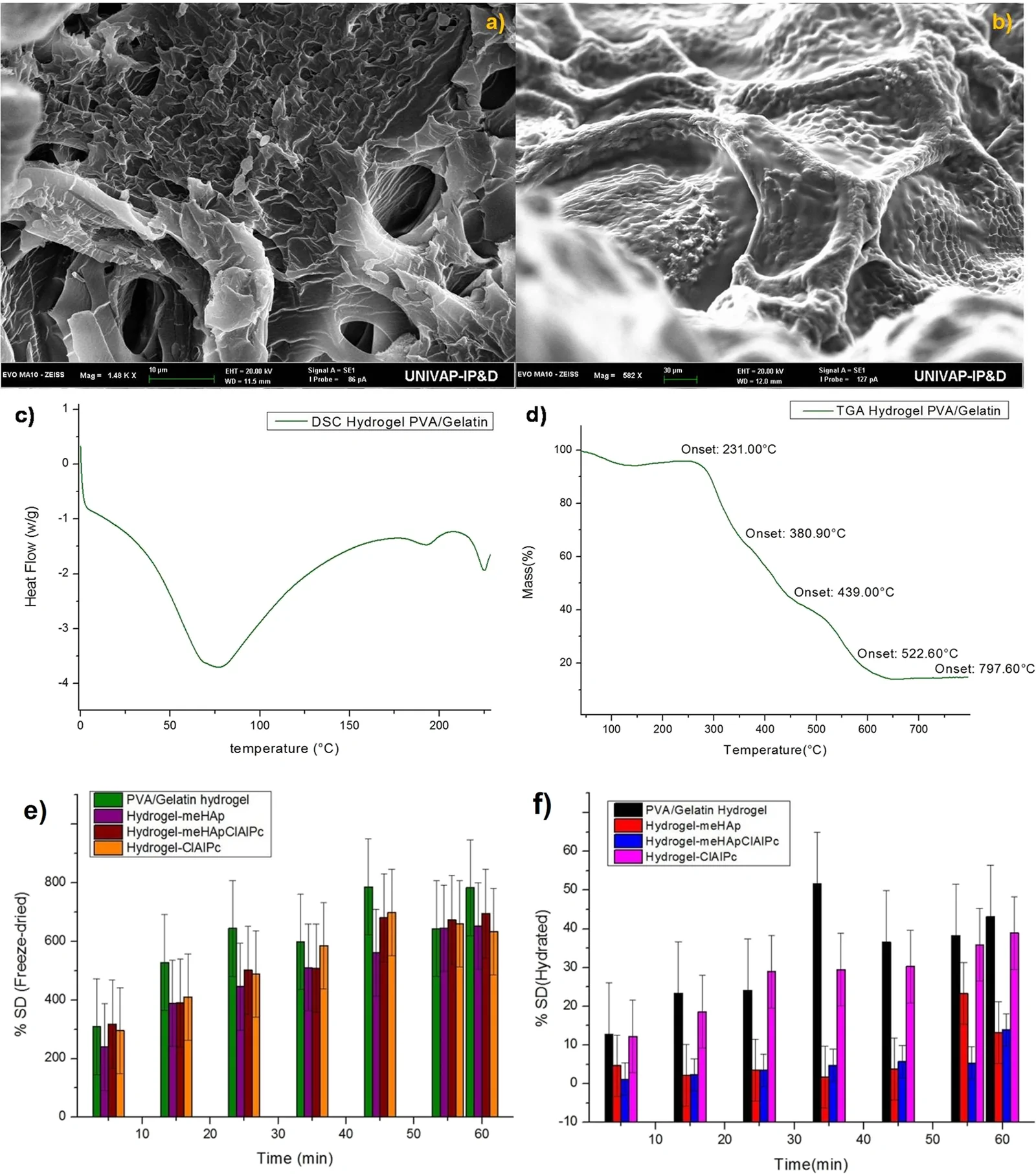
Samples characterization: (a) SEM micrograph of hydrogel PVA/gelatin, (b) SEM micrograph of meHAp hydrogel, (c) TGA of hydrogel PVA/gelatin, (d) DSC of hydrogel PVA/gelatin, (e) % SD of freeze-dried samples, (f) %SD hydrated samples.

Figure 1b allows for the observation of spherical meHAp particles dispersed across the surface of the synthesized hydrogel. As noted in the Experimental Section, the preliminary characterization of the meHAp is presented in Ambrosio et al.^19^ The size of the meHAp incorporated into the hydrogel matrix, measured by DLS, was 102.5 nm with a PdI of 0.414, and images from the previously published manuscript demonstrate the spherical morphology of the meHAp formulations. According to Silva-Holguín and Reyes-López,^27^ the size of the formulations, as well as their morphology, can influence the material’s diffusion potential and, consequently, its therapeutic capacity.

According to Luo et al.,^28^ particles with an average size of 100 nm are easily transported through the bloodstream due to the EPR effect (enhanced permeability and retention effect). According to Chan,^29^ the main process of entry of formulations into cells is passive diffusion, facilitated via EPR effect, which occurs when, during the tumor growth process, irregular blood vessels are formed that have gaps, allowing the entry of DDS.

### 2.2 Thermal Characterization

Thermal studies: TGA (Figure 1c) shows 5 mass-loss regions at 231.00 °C, 380.90 °C, 439.00 °C, 522.60 °C, and 797.60 °C. DSC (Figure 1d) allows observing the system’s behavior when exposed to high temperatures, including mass loss and changes in enthalpy, and shows two regions of endothermic events, namely at 67.63 and 224.55 °C.

The DSC curve (Figure 1c) shows two endothermic events at 67.63 and 224.55 °C. The first event is related to the glass transition temperature of gelatin, and the other to the melting temperature of PVA.^25,26^

The TGA thermogram (Figure 1d) allows the observation of 5 regions of mass loss, located at 231.00 °C (referring to the removal of mixture from the polymer chain), 380.90 and 439.00 °C (referring to the removal of OH groups of the PVA chain and degradation of proteins present in the gelatin chain) and 522.60 and 797.60 °C (referring to the degradation of the PVA chain).^24,25^

Both analyses present characteristics of both gelatin and PVA, which can confirm their mixture and, consequently, the characteristics incorporated into the hydrogel chain formed.

### 2.3 Swelling Capacity

The swelling capacity of hydrogel is essential for the encapsulation process and for its future use as a DDS. The swelling capacity of hydrogel groups is shown on Figure 1e (freeze-dried samples) and Figure 1f (hydrated samples) and described in Table 1.

**Table 1:** %SD Capacity of Hydrogel Groups^a^

hydrogel group	%SD hydrated	%SD freeze–dried
ClAlPc hydrogel	27.76	539.07
meHAp hydrogel	7.46	492.16
meHApClAlPc hydrogel	5.23	538.47
PVA/gelatin hydrogel	32.8	613.3

a Source: the authors.

The swelling capacity of hydrogels enables these systems to absorb water or solvents of interest without dissolving the polymer. Chain cross-linking, pore size, pH, temperature, and chain composition influence swelling capacity.^16,30,31^

The swelling process of a hydrogel system occurs in three stages: surface hydration (where water molecules first interact with the polar components of the hydrogel chain), followed by the interaction of the hydrogel with water molecules, and finally, the accumulation of water within the hydrogel chain.^32^

The freeze–dried PVA/gelatin hydrogel presented 613.3% of sweeling degree, compared with literature Labus et al.^33^ reported an average swelling degree of 626% for a hydrogel formulation containing 2% lyophilized PVA over a 24 h test at 30 °C.

According to Lopes et al.,^34^ lyophilization contributes to increasing the porosity of the hydrogel—corroborating the data that demonstrate greater swelling capacity—and ensures greater stability of the formulation against microbial contamination, which represents a fundamental challenge in relation to the storage and stability of hydrogel systems, which can be observed by comparing the formulations in their two modes.

The highest degree of swelling was observed in the empty hydrogel sample, followed by the hydrogel encapsulated with ClAlPc, which, despite being dispersed along the chain, occupies some space and prevents complete swelling compared to the empty hydrogel. The two formulations containing lower amounts of meHAp exhibit higher swelling rates as the chain becomes denser. According to Mohammadpour et al.^35^ and Manzoor et al.,^36^ the accumulation of HAp along the chain causes it to contract and exhibit reduced mobility, thereby decreasing the size and availability of pores.^16,35^ According to Słota et al.,^37^ the addition of ceramic components to the matrix of systems reduces the rate and capacity for fluid absorption and release. In addition to the composition and morphology of the chain, the degree of cross-linking of the system also affects the degree of swelling of the material, since the greater the cross-linking, the more rigid the chain and the more difficult its expansion process.^38^

Morphological and swelling analyses further support these findings. The incorporation of meHAp reduced the mobility of the polymer chains, thereby limiting network mobility and reducing the free volume available for drug diffusion. The reduction in the swelling degree observed in the hybrid system directly correlates with the lower release rates, as the denser matrix restricts the influx of medium and the subsequent efflux of the drug.^16,35,36^

The correlation between SEM observations and swelling behavior confirms the formation of a dense hybrid matrix. The reduction in porosity observed via SEM directly explains the lower swelling rates, as there is less free volume available for water penetration. This synergy is a key design feature of our engineered scaffold: the meHAp microspheres serve a dual purpose, acting as both structural reinforcers that stabilize the hydrogel and as secondary reservoirs that modulate the release of ClAlPc by limiting solvent-mediated diffusion.^16,35,36^

### 2.4 Fourier Transformed Infrared Spectroscopy

Figure 2 shows the bands corresponding to the hydrogel samples synthesized together with their formers for comparison. All bands are described in Tables 2, 3, and 4.

**Figure 2:**
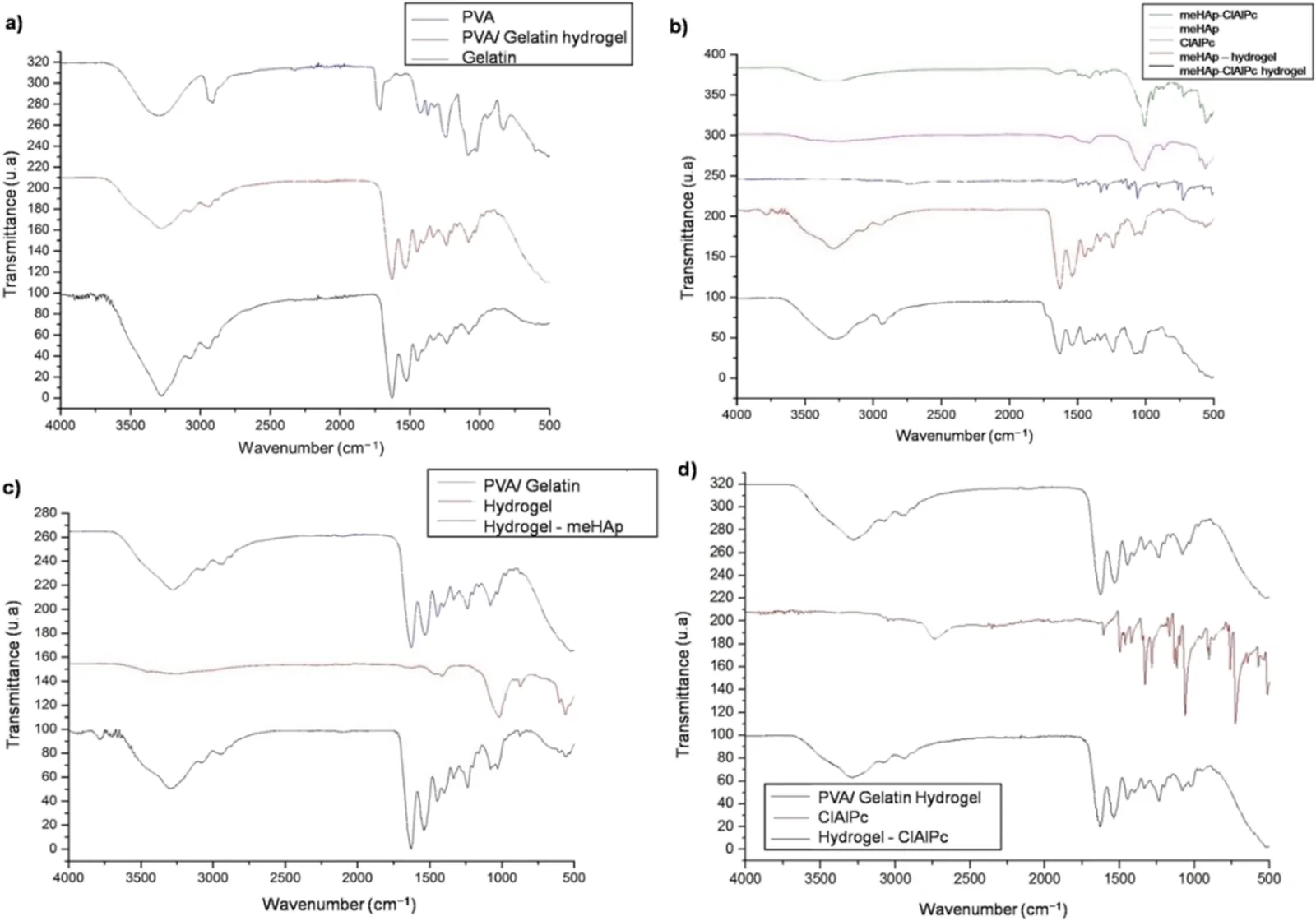
FTIR spectra of: (a) PVA, gelatin, and hydrogel PVA/gelatin, (b) meHApClAlPc, meHAp, ClAlPc, hydrogel meHAp, hydrogel meHApClAlPc, (c) PVA/gelatin hydrogel, meHAp, hydrogel meHAp, and (d) PVA/gelatin hydrogel, ClAlPc, and ClAlPc hydrogel.

**Table 2:** Bands Attributions of Gelatin, PVA, and PVA/Gelatin Hydrogel^a^

band	gelatin	PVA	hydrogel
C–H bending	1332 cm^–1^	1376 cm^–1^	1338 cm^–1^
C–H stretching	2936 cm^–1^	2940 cm^–1^	2939 cm^–1^
C–N stretching	1236 cm^–1^	1243 cm^–1^	1234 cm^–1^
C–O stretching	1635 cm^–1^	1083 cm^–1^	-
	-	1715 cm^–1^	-
C–O–C bending	-	1424 cm^–1^	-
N–H bending	548 cm^–1^	-	1081 cm^–1^
	1074 cm^–1^	-	1531 cm^–1^
	1522 cm^–1^	-	
O–C–O stretching (symmetrical)	1437 cm^_1^	-	1400 cm^–1^
	–	-	
O–H stretching	3288 cm^–1^	3299 cm^–1^	3063 cm^–1^
	^–^	-	3284 cm^–1^

a Source: the authors (adapted from SALAHELDIN, H et al.,^39^ Hassan, N et al.,^40^ Tsioptsias, C. et al.,^41^ Kovtun, G. et al.,^42^ A. Mohammed et al.^43^ and Chi, H. et al.^44^)

**Table 3:** Bands Attributions of meHAp, meHApClAlPc and ClAlPc^a^

band	meHAp	meHApClAlPc	ClAlPc
C–C stretching	-	-	1608 cm^–1^, 1498 cm^–1^
	-	-	1461 cm^–1^, 1421 cm^–1^
	-	-	1329 cm^–1^
C–H bending	-	-	1285 cm^–1^, 1186 cm^–1^, 1166 cm^–1^, 1133 cm^–1^, 1018 cm^–1^, 1095 cm^–1^, 1061 cm^–1^
	-	-	
	-	-	
	-	-	
C–H bending (out-of-plane)	-	756 cm^–1^, 723 cm^–1^	760 cm^–1^, 727 cm^–1^
	-		
C–O bend	870 cm^–1^	897 cm^–1^, 868 cm^–1^	-
C–O stretching	-	1648 cm^–1^, 1497 cm^–1^	-
	-	1465 cm^–1^	-
C–O symmetric stretching	1638 cm^–1^	-	-
	1467 cm^–1^	-	-
	1411 cm^–1^	-	-
O–P–O bond stretching vibration	-	1406 cm^–1^	-
	-	^–^	-
P–O symmetric stretching	1017 cm^–1^	1006 cm^–1^, 596 cm^–1^	-
	602 cm^–1^	554 cm^–1^	-
	559 cm^–1^	-	-
rocking vibration of the AlPc core	-	948 cm^–1^	909 cm^–1^
	-	-	-

a Source: the authors (adapted from Longkaew et al.,^45^ Song et al.,^46^ Darshana Senarathna et al.,^47^ Ye et al.,^48^ Wang et al.,^49^ Yao et al.,^50^ and Zubieta-Otero et al.^51^).

**Table 4:** Bands Attributions of meHAp Hydrogel, meHApClAlPc Hydrogel and ClAlPc Hydrogel^a^

band	meHApClAlPc hydrogel	meHAp hydrogel	ClAlPc hydrogel
amide II	-	1331 cm^–1^	-
amide III	-	-	1237 cm^–1^
	-	-	1202 cm^–1^
C–C stretching	-	-	1450 cm^–1^
C–H bending	1330 cm^–1^	1235 cm^–1^	1538 cm^–1^
		2942 cm^–1^	1204 cm^–1^
C–H stretching	2927 cm^–1^	-	2945 cm^–1^
C–O stretching	1632 cm^–1^	1541 cm^–1^	1083 cm^–1^
	1448 cm^–1^	1448 cm^–1^	-
	1375 cm^–1^	-	-
C–O stretching (asymmetric)	-	1402 cm^–1^	1400 cm^–1^
	-	-	-
N–H bending	-	-	1538 cm^–1^
	-	-	1024 cm^–1^
O–H bending	-	1624 cm^–1^	1636 cm^–1^
O–H stretching	3279 cm^–1^	3295 cm^–1^	3277 cm^–1^
	^–^	3071 cm^–1^	3058 cm^–1^
P–O symmetric stretching	1065 cm^–1^	1079 cm^–1^	-
	-	1029 cm^–1^	-
vibration of the AlPc core	948 cm^–1^	-	955 cm^–1^
	-	-	-

a Source: the authors (adapted from Longkaew et al.,^45^ Song et al.,^46^ Darshana Senarathna et al.,^47^ Ye et al.,^48^ Wang et al.,^49^ Yao et al.,^50^ and Zubieta-Otero et al.^51^).

The comparison of the assignments related to gelatin and PVA with the assignments related to the empty hydrogel allows (Table 2, Figure 2a) the observation that the final structure of the hydrogel has greater similarity to the chemical structure of gelatin, since the PVA chain is simpler and presents the same functional groups found in gelatin (CH_2_, CH, and OH).

Table 3 presents data demonstrating the encapsulation of the meHAp formulation with ClAlPc, resulting in the meHApClAlPc formulation, as evidenced by the band at 948 cm^–1^, assigned to the ClAlPc core (Figure 2b).

Table 4 presents the assignments related to the three encapsulated hydrogel formulations, which allows the observation of the presence of the 1411 cm^–1^ band in the formulations containing meHAp (referring to the carbonate ions of the amorphous phase of meHAp) and the band of approximately 948 cm^–1^ (referring to the ClAlPc core) (Figure 2b,c,d).

### 2.5 UV–Visible, Photosensitizer Release Studies and Encapsulation

The spectra (Figure 3a,b) show that the spectral profile of ClAlPc did not change when in contact with meHAp, hydrogel meHApClAlPc, or hydrogel ClAlPc. Figure 3c show the hydrogel PS release study, and the difference between ClAlPc hydrogel and meHApClAlPc hydrogel.

**Figure 3:**
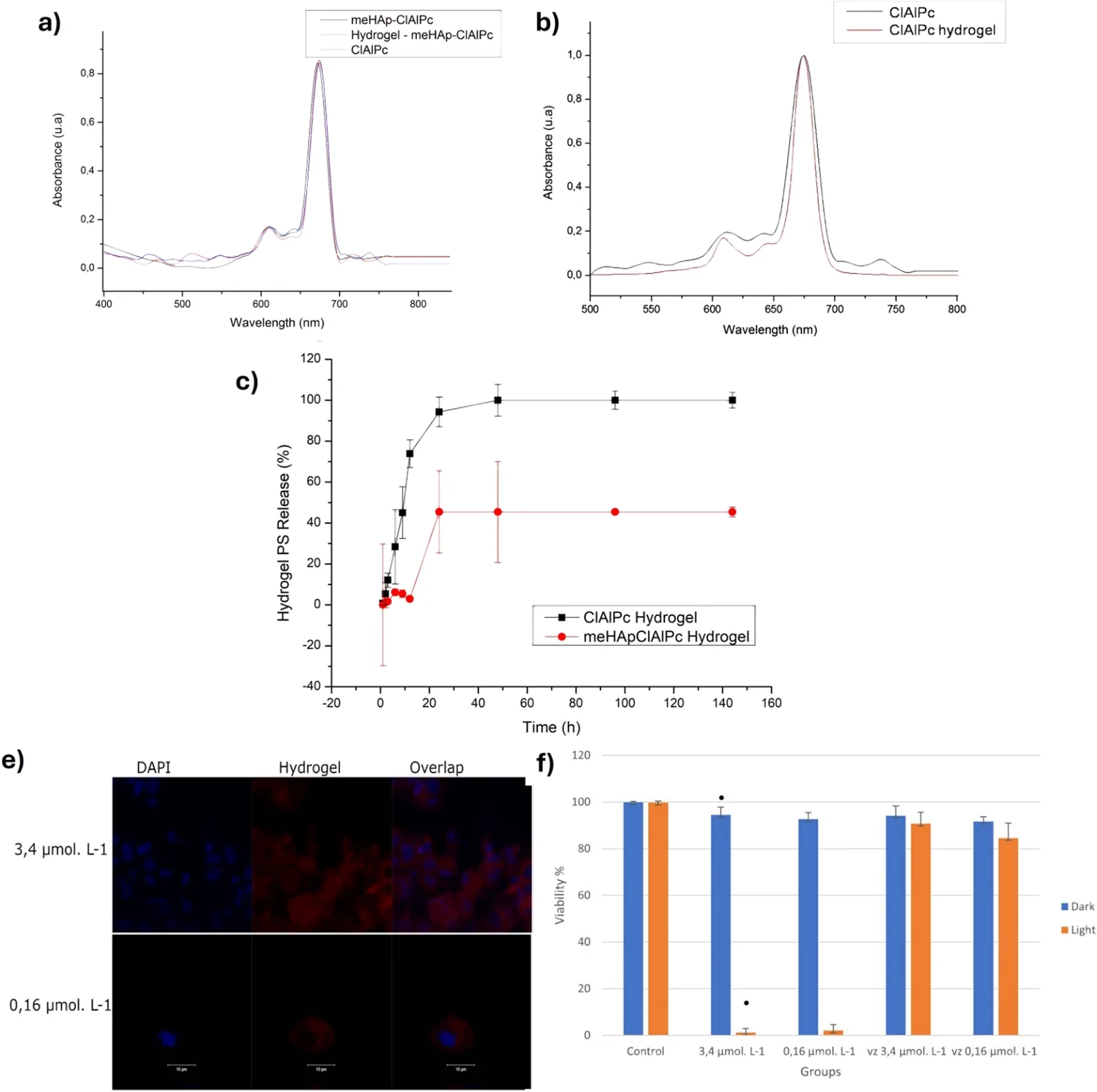
(a) UV–visible spectra of meHApClAlPc, hydrogel meHApClAlPc and ClAlPc, (b) UV–visible spectra of ClAlPc and hydrogel ClAlPc, (c) hydrogel PS release with standard deviation, (d) DAPI, (e) viability studies.

The meHApClAlPc sample showed a maximum absorbance peak at 672 nm, while the ClAlPc, ClAlPc hydrogel, and meHApClAlPc hydrogel samples showed a maximum absorbance of 674 nm (all curves presented underwent a normalization process). According to Moiseeva et al.,^52^ the maximum absorption peak of phthalocyanines is in the spectral range between 600 and 700 nm. These compounds are widely used in PDT applications due to their high capacity to generate reactive oxygen species.

The irradiation source had a nominal wavelength of 660 nm, whereas the ClAlPc-containing formulations exhibited absorption maxima at approximately 672–674 nm. Although 660 nm remains within the Q-band absorption region and was sufficient to produce a photoinduced biological response, it does not correspond exactly to the measured absorption maximum. Irradiation closer to 672–674 nm could potentially increase photon absorption; however, a direct wavelength comparison was not performed in this study.

Figure 3a,b shows the UV–visible spectrum of the samples, which shows no changes compared to the isolated PS sample, indicating that ClAlPc remained in its monomeric form and without the occurrence of the aggregation process after encapsulation, one of the prerequisites for the application of the system in protocols involving PDT.^53^

The results of the release tests (Figure 3c) show the same pattern when comparing the meHApClAlPc hydrogel with the ClAlPc hydrogel, since the hydrogel containing only PS shows almost twice the release, considering the same time interval. The release values found were 94.32% (ClAlPc hydrogel) and 45.6% (meHApClAlPc hydrogel).

Release assays revealed a distinct kinetic profile between the formulations: while the hydrogel containing free PS (ClAlPc hydrogel) released 94.32% of the drug, the microsphere-incorporated formulation (meHApClAlPc hydrogel) exhibited an average release of only 45.6% within the same time frame. This nearly 50% reduction underscores the pivotal role of meHAp in modulating the delivery profile, suggesting a more sustained and prolonged therapeutic window. This superior release control can be attributed to a dual-barrier mechanism: the photosensitizer must first be released from the hydroxyapatite microspheres before diffusing through the hydrogel’s polymeric network.^54,55^

Release from a polymeric matrix depends on factors such as pore or particle size and PS solubility. According to Lu et al.,^56^ the release process can occur by 3 means: diffusion (the PS leaves the polymeric matrix to the outside), swelling (the matrix absorbs water and during the expansion process releases the PS), and erosion (as the chain dissolves, the PS is released). In the case analyzed, the authors observed some structural changes at the hydrogel polymeric matrix, but without specific release process detected.

Regardless of the release process used, in the formulation containing meHApClAlPc, the PS goes through the meHAp release process and then, from the hydrogel, that is, the PS goes through the process twice, while in the hydrogel containing ClAlPc, the PS only needs to be released from the hydrogel matrix.

Chandna et al.^57^ tested the release of lignin hydrogels (in PBS medium) containing Rose Bengal and silver nanoparticles and obtained a maximum release of 28%. Xiong et al.^58^ obtained a maximum release of 36.9% testing the release of hydrogels containing Nile blue in PBS. Glass et al.^59^ obtained a release rate of 38% of porphyrin-based drugs in hydrogels.

The encapsulation process for the PVA/gelatin hydrogel was carried out for ClAlPc, meHAp, and meHApClAlPc. In cases where the hydrogel contained only ClAlPc or only meHAp (without PS), the encapsulation process was performed simply by dispersing the active agent of interest into the formulation, as described in the methodology. For the encapsulation of meHAp within the hydrogel (forming the dual-barrier system), the system previously described by Ambrosio et al.^19^—consisting of meHAp encapsulated with ClAlPc—was dispersed into the hydrogel matrix, resulting in the formation of the meHApClAlPc hydrogel. These processes yielded encapsulation efficiencies (EE) of 98.64% (ClAlPc hydrogel) and 99.99% (meHApClAlPc hydrogel). Although the process consists merely of mixing, mechanical agitation can, in some instances, leave undissolved fractions following gelation.

### 2.6 Qualitative Fluorescence Image of the Cell-Associated Formulation

The synthesized systems (meHAp hydrogel and meHApClAlPchydrogel) were evaluated in vitro using a gliosarcoma cell culture. Following hydrogel administration, a swelling process occurs, characterized by the gradual expansion of polymer chains. Depending on the material’s physicochemical properties and composition, the matrix may also undergo progressive degradation.

In responsive hydrogels, external stimuli—such as temperature and pH variations—can promote the expansion of the polymer network during swelling. An increase in mesh size (the free spaces within the hydrogel network) enables the release of the encapsulated photosensitizer (PS). When the mesh size exceeds the dimensions of the PS, diffusion becomes the primary release mechanism, acting in conjunction with the dynamics of the polymer matrix.^60^ Thus, release kinetics are governed by the relationship between the hydrogel mesh size and the dimensions of the photosensitizer.^61^

In the system developed in this study, it is expected that, upon release into the extracellular medium, ClAlPc will be internalized by the cells—likely via passive diffusion and/or endocytic pathways—given that no additional functionalization or targeting strategies were employed for the hydrogel.^61^ Consequently, the proposed system acts as a local reservoir for photosensitizer release within the tumor microenvironment, rather than relying on accumulation mediated by the EPR effect.

Fluorescence microscopy revealed red fluorescence associated with cells exposed to both formulations after 30 min of incubation. A visually stronger signal was observed for the ClAlPc hydrogel condition than for the meHApClAlPc hydrogel condition. However, because the formulations were tested at different concentrations and fluorescence intensity was not quantitatively measured, the images do not permit a direct comparison of cellular uptake. In addition, overlap between the red and DAPI channels in two-dimensional images should not be interpreted as definitive evidence of nuclear localization.

The lower fluorescence intensity observed for the meHApClAlPcformulation may be related to the system’s composition and the arrangement of hydroxyapatite microspheres on the hydrogel surface—factors that potentially influence the release and, consequently, the availability of the photosensitizer for internalization. However, this hypothesis requires confirmation through specific release and cellular uptake studies. In this context, quantitative experiments using flow cytometry, spectrofluorimetry, or image-based fluorescence analysis will be necessary in future investigations.

According to Chen et al.,^62^ a promising strategy for the local administration of hydrogels associated with photodynamic therapy (PDT) involves the use of injectable formulations applied before the material fully gels. This approach facilitates direct administration to the tumor site and may enhance the local retention of the photosensitizer.

Cell viability was assessed using the trypan blue exclusion test, which relies on the ability of viable cells to exclude the dye. In contrast, cells with compromised plasma membranes lose this selectivity and accumulate the dye intracellularly. The assays were conducted in the presence and absence of light irradiation, allowing for the evaluation of the system’s phototoxic potential. In the absence of light, the cellular viability values are high at both concentrations tested, indicating reduced cytotoxicity of the compound in the dark. This result demonstrates that the formulation exhibits low basal toxicity and that its cytotoxic activity depends on light activation—an undesirable characteristic for photodynamic therapy applications.

### 2.7 Photodynamic Therapy Protocol

The advantages of using a hydrogel as a modified-release system include protection of active agents, increased formulation half-life, and reduced degradation of active ingredients. In addition to these properties, combining a hydrogel system with a particulate component further enhances them, thereby favoring treatment outcomes.^63^

The application of PDT proves to be a promising strategy in the treatment of aggressive tumors, such as gliosarcoma. The graphs (Figure 3e) show that, at the concentrations tested, cell viability was reduced by 98.79% for hydrogel ClAlPc and 97.94% for hydrogel meHApClAlPc after irradiation. In the dark group, the reduction in viability for both concentrations was less than 10%. In the groups exposed to the empty hydrogel, there was no significant reduction in cell viability, demonstrating that the nanoparticle was not cytotoxic.

The marked difference between the PDT and dark groups is consistent with a light-dependent photodynamic effect. However, ROS generation was not directly measured, and the present results do not identify the reactive species or molecular pathways responsible for cell damage. Future studies should include probes for total intracellular ROS and, where appropriate, singlet oxygen or other specific photoproducts.

It is important to highlight that, in the control groups, the compounds containing PS and the groups subjected only to irradiation without PS showed a reduction in cell viability of less than 10%. These data reinforce the notion that the observed cytotoxicity is directly related to the activation of PS by light, using a nonpolymeric nanogel based on toluene, loaded with Rose Bengal and hypericin, Torres-Martínez et al.^64^ obtained an approximately 80% reduction in cell viability in HT-29 cells (colorectal adenocarcinoma). Regarding the presence of hydroxyapatite (tested in both the empty control group and the group loaded with meHAp), Sarita et al.^65^ demonstrated, through tests with 3-(4,5-dimethylthiazol-2-yl)-2,5-diphenyltetrazolium bromide (MTT), that chitosan, hydroxyapatite, and hyaluronic acid hydrogel formulations were biocompatible and nontoxic to J774 macrophage cultures. Sumin et al.^66^ tested ammonia-doped manganese-hydroxyapatite nanoparticles for the treatment of MDA-MB-231 cells (triple-negative breast cancer) and obtained a 10% reduction in cell viability at a concentration of 50 μg mL^–1^ and a 17% reduction in viability when testing a concentration of 250 μg mL^–1^.

Cell viability was assessed only by Trypan Blue exclusion. As this assay mainly reflects membrane integrity, the results should be interpreted as an initial estimate of photoinduced cytotoxicity. Confirmation using an orthogonal metabolic or fluorescence-based assay will be required in future studies.

From a biomedical engineering perspective, the controlled release provided by meHAp offers a strategic advantage. Delivery systems characterized by rapid ‘burst release’ often lead to unnecessary toxicity or premature drug degradation. The combination of a 3D hydrogel matrix with a particulate ceramic component (meHAp) enhances therapeutic outcomes by ensuring drug protection and allowing photodynamic therapy (PDT) to be performed more efficiently and localized at the tumor bed.

Because no nonmalignant cell model was included, the study does not establish tumor selectivity or cytocompatibility with healthy neural or stromal tissues.

The present results should be interpreted within the exploratory scope of the study. Although incorporation of meHAp was associated with a lower cumulative release of ClAlPc from the hydrogel, the release of ClAlPc from meHAp microspheres alone was not evaluated, and the release data were not fitted to kinetic models. Therefore, the individual contributions of microsphere desorption, hydrogel diffusion, swelling, and matrix erosion cannot be established. In addition, the biological formulations were evaluated at different total material concentrations and should not be interpreted as a dose-matched head-to-head comparison. Finally, the 30 min incubation protocol represents an initial photodynamic activity assay and does not establish that prolonged release improves PDT efficacy.

Only one preirradiation incubation period and one light dose were evaluated. Therefore, the experiment demonstrates photoactivity under a specific protocol but does not establish an optimized treatment condition or determine whether prolonged release from the meHAp-containing formulation improves PDT outcome. Future studies should evaluate multiple incubation periods, fluences, irradiances, and photosensitizer-equivalent concentrations.

### 2.8 Statistical Analysis Statement

All tests were performed in triplicate, and all data were submitted to ANOVA followed by Tukey’s test multiple-comparisons test. *p < 0.05 compared with the corresponding control; additional symbols and comparisons are defined in the figure. Microsoft Excel was used to generate charts. All data are presented as the mean ± standard deviation (SD) of three independent experiments.

## 3 Conclusion

A PVA/gelatin hydrogel containing ClAlPc-loaded hydroxyapatite microspheres (meHAp) was developed and physicochemically characterized. The incorporation of meHAp altered the hydrogel’s morphology and swelling behavior and was associated with reduced cumulative ClAlPc release under the conditions evaluated. Our results demonstrate that meHAp incorporation not only imparts structural integrity to the matrix but also modulates ClAlPc release via a dual-barrier mechanism, preventing burst release. The system achieved encapsulation efficiencies of 98.64% for the ClAlPc-loaded hydrogel formulation and 99.99% for the meHAp–ClAlPc hydrogel formulation. Tests involving gliosarcoma cell cultures showed a reduction in cell viability of 98.79% for the ClAlPc hydrogel and 97.94% for the meHAp–ClAlPc hydrogel, demonstrating that this system is a highly effective tool for Photodynamic Therapy. Preliminary experiments on 9L/lacZ cells demonstrated reduced effects in the absence of light and a marked loss of viability following irradiation for both tested formulations containing the photosensitizer. However, biological conditions were not standardized regarding dosage, and only one tumor cell line, one irradiation condition, one incubation period, and one viability assay method were evaluated. Therefore, the results support the material as a proof-of-concept platform for future investigations but do not establish therapeutic superiority, selectivity, biocompatibility, intracranial safety, or clinical efficacy.

## 4 Experimental Section

### 4.1 Materials

Hydrochloric acid (HCl), dimethyl sulfoxide (DMSO), and Paraformaldehyde were purchased from Dinâmica, Química contemporânea Ltd.a. Poly­(sodium 4-styrenesulfonate) (PSS), Trypan Blue, and Gelatin were purchased from Sigma-Aldrich. PVA was purchased from Vetec Química fina Ltd.a. Sodium carbonate (Na_2_CO_3_), sodium phosphate (Na_2_HPO_4_), and ethanol were purchased from Labsynth Ltd.a. Chloroaluminum phthalocyanine (ClAlPc) was purchased from ACROS Organics. Calcium chloride (CaCl_2_) was purchased from QEEL–Química Especializada Erich Ltd.a. Dulbecco’s modified eagle medium (DMEM) was purchased from Gibco, penicillin/streptomycin was purchased from LGC Biotechnology. ProLong and 4′,6-diamidino-2-phenylindole (DAPI) were purchased from Thermofisher.

### 4.2 Hydroxyapatite Microspheres Synthesis

The entire process for the synthesis and characterization of the hydroxyapatite microspheres used in this study was previously described in a prior paper by the research group (Ambrosio et al.^19^). The present study focuses solely on the hydroxyapatite microspheres (meHAp) and the ClAlPc-encapsulated hydroxyapatite microspheres (meHApClAlPc). In this study, the meHApClAlPc microspheres were incorporated into a PVA/gelatin hydrogel to develop the proposed dual-barrier release system. Therefore, the focus of this work is the preparation and evaluation of the hydrogel-based composite system.

In summary, hydroxyapatite microspheres were prepared using a vaterite template. Vaterite was synthesized using CaCl_2_, PSS, and Na_2_CO_3_ precursors. For the synthesis of hydroxyapatite microspheres, the hydrothermal method was used; the synthesized vaterite microspheres were mixed with a Na_2_HPO_4_ solution and placed in an autoclave for 15 min at 120 °C. After cooling, the white precipitate was separated by centrifugation, then washed and dried at 60 °C for 24 h.

To encapsulate the meHAp samples with ClAlPc, a 5 mL solution of ClAlPc (50 mg mL^–1^), ethanol, and DMSO was prepared. The microspheres were in contact with the PS solution in a closed system for 24 h.

### 4.3 Hydrogel Synthesis

PVA/gelatin hydrogels were prepared according to the method described by Imtiaz et al.,^20^ with a few modifications. Briefly, PVA was added to 100 mL of deionized water at 80 °C for 1h under constant stirring to obtain dissolution. The resulting homogeneous transparent aqueous solution was mixed with gelatin (1:1 proportion with PVA) and 0.05 mL of HCl (35%). The solution was stirred at 70 °C to carry out esterification between PVA and gelatin. Subsequently, it was placed in an ultrasound for 1 h to homogenize and remove bubbles. The solution was then gelled at room temperature. The sample was then washed in three centrifugation cycles (20 min, 4000 rpm) to remove impurities, and the drying process was subsequently carried out at approximately 40 °C.

The encapsulation process of hydroxyapatite microspheres (meHAp and meHApClAlPc) and ClAlPc in the synthesized hydrogel was carried out in the last stage of the synthesis after the addition of HCl to the polymeric mixture of PVA and gelatin.

The hydrogel samples were characterized by scanning electron microscopy (SEM), Fourier transformed Infrared spectroscopy (FTIR), thermogravimetry (TGA), differential scanning calorimetry (DSC), swelling degree (% SD), UV–visible spectroscopy, encapsulation efficiency (EE), PS release, and *in vitro* studies.

### 4.4 Scanning Electron Microscopy

The morphology of the synthesized hydrogel was analyzed by scanning electron microscopy using the equipment EVO-MA10 (Zeiss, Germany). The three-dimensional network morphology is one of the main characteristics of a hydrogel system.

### 4.5 Fourier-Transformed Spectroscopy

The FTIR spectra were collected in attenuated total reflectance mode using a Fourier transform FTIR spectrophotometer (PerkinElmer Spectrum 400, USA), equipped with an attenuated total reflectance accessory with a diamond crystal. The spectra were obtained over 4000–450 cm^–1^, with a spectral resolution of 4 cm^–1^ and an average of 32 scans/point. The background was made with clean crystals in an ambient atmosphere. The samples analyzed were hydrogel PVA/gelatin, gelatin, PVA, hydrogel meHApClAlPc, hydrogel ClAlPc, hydrogel meHAp, ClAlPc, meHAp and meHApClAlPc.

### 4.6 Thermogravimetry and Differential Scanning Calorimetry

Both tests were carried out using the STA 449 F3 Jupiter (NETZSCH) equipment. The TGA allowed the evaluation of the system’s mass loss as a function of the temperature at which the sample was exposed. The DSC technique enabled the evaluation of the intrinsic properties of the materials that comprise the studied system. The analyses were carried out using samples of the PVA/gelatin hydrogel.

### 4.7 Swelling Degree

The presence of three-dimensional networks in their morphology gives hydrogel systems the ability to swell, i.e., to absorb and retain solvents without the hydrogel chains dissolving. The treatment of the test samples was based on the methodology described by Saidi et al.^67^ Initially, the samples were weighed and immersed in deionized water for a pre-established period. After the determined period, the samples were dried by pressing on absorbent paper and weighed again. All were carried out in triplicate, and the degree of swelling was calculated as follows on eq 1

1 %SD=(massoftheswollensystem−Iinitialmass)/(initialmass)×100

### 4.8 UV–Visible Spectroscopy

UV–visible studies were performed using a DeNovix DS-11+ spectrophotometer, which allowed comparison of the spectra of the meHApClAlPc hydrogel, meHApClAlPc, ClAlPc hydrogel, and ClAlPc samples, and assessment of the occurrence or absence of spectral changes due to the encapsulation process. All graphs were normalized to the maximum intensity/absorbance for ease of visualization.

### 4.9 Encapsulation Efficiency

For encapsulation efficiency, formulations containing PS (ClAlPc hydrogel and meHApClAlPc hydrogel) were portioned and immersed in 4 mL of DMSO solution (10% in ethanol) for 24 h. After this period, the supernatants were analyzed using a DeNovix DS-11+ spectrophotometer. The EE calculation (eq 2) was performed according to the method described by Asad et al.^21^ Based on discovery 2, Lambert–Beer’s law, and the discovery curve described in Ambrosio et al.^19^

2 $$EE=\frac{total\;PS\;added\;to\;the\;formulation-PS\;present\;in\;the\;supernatant}{total\;PS\;added\;to\;the\;formulation}\times 100$$

### 4.10 Photosensitizer Release Studies

The evaluation of the modified release from encapsulated hydrogel samples (ClAlPc hydrogel and meHApClAlPc hydrogel) was performed using adaptations of the methodologies described by Bakhshi et al.^68^ and Fernandes.^69^

For the study, preportioned hydrogel samples were placed in 10 mL of PBS, and the samples were kept in an incubator at a controlled temperature of approximately 37 °C. At predetermined intervals (3, 6, 9, 12, 24, 48, 96, and 144 h), 3 mL aliquots of PBS were withdrawn from the reaction medium, and 3 mL of PBS was immediately added to replenish and restore the volume to 10 mL throughout the study period. The withdrawn aliquots were analyzed via ultraviolet–visible spectroscopy using a DeNovix DS-11+ instrument. The tests were performed in triplicate.

### 4.11 *In vitro* Studies

Cells of the 9L/LacZ cell line (gliosarcoma) were cultured in DMEM supplemented with 10% fetal bovine serum (FBS) and 1% Penicillin/Streptomycin and kept in an incubator at 37 °C with 5% CO_2_. Two groups were used for the tests: hydrogel ClAlPc (3400 μg mL^–1^) and hydrogel meHApClAlPc (160 μg mL^–1^). Internalization studies and application of the PDT protocol were performed.

### 4.12 Cell Internalization

Initially, the cells adhered to glass coverslips distributed in 24-well plates and incubated for 24 h in an incubator at 37 °C, 5% CO_2_. After this period, the culture medium was removed. The analyzed formulation was then added and incubated for 30 min, followed by washing with PBS (phosphate buffered saline) and fixation with 4% paraformaldehyde for 15 min at room temperature. The slides were mounted with ProLong Diamond Antifade Mountant with DAPI (4′,6-diamidino-2-fenilindol) and analyzed under a Zeiss—LSM 700 confocal microscope (λ_exc._ DAPI = 405 nm, λ_exc._ formulation = 555 nm).

### 4.13 Photodynamic Therapy

#### 4.13.1 Experimental Groups

The cells were separated into groups: control—no treatment; dark—incubated with formulation and kept in the dark; light—cells subjected only to irradiation; PDT—incubated with formulation and irradiated.

The cells were transferred to a 96-well plate at a density of 1 × 10^5^ cells per well. After 24 h of cell adhesion, the systems were added at the concentrations described in the previous item and incubated for 30 min in the oven at 37 °C and 5% CO_2_. After this period, the formulation was removed, and PBS was added. One plate was kept in the dark, and the other was irradiated. After the PDT application, the PBS from both plates was removed, and the DMEM medium was added. The plates remained at 37 °C and 5% CO_2_ for 24 h before analysis. The experiment was performed in triplicate, and the groups were protected from light throughout. After the treatments, viability was assessed using the Trypan Blue exclusion method. Viability was assessed at a single 24 h time point using Trypan Blue exclusion. Therefore, the results reflect an early membrane-integrity-based response and do not establish long-term reproductive cell death.

#### 4.13.2 Irradiation

A Biotable (Biopdi/Irrad-Led) with 54 LEDs was used for irradiation. Its irradiance was 36.8 mW cm^–2^ at 660 ± 5 nm, and its fluence was 10 J cm^–2^.

#### 4.13.3 Cell Viability Analysis

Cell viability analysis was performed 24 h after PDT application using a 0.2% Trypan Blue solution and a 5 min incubation. After this time, the solution was removed, and PBS was added. Subsequently, images of 10 different random fields were captured for each well using an inverted microscope (Zeiss Axio Vert. A1). Cell counts were performed using ImageJ software (Plugins—Analyze—Cell Counter), and the tests were performed in triplicate.
